# An annotated corpus of clinical trial publications supporting schema-based relational information extraction

**DOI:** 10.1186/s13326-022-00271-7

**Published:** 2022-05-23

**Authors:** Olivia Sanchez-Graillet, Christian Witte, Frank Grimm, Philipp Cimiano

**Affiliations:** Semantic Computing Group, Cluster of Excellence Cognitive Interaction Technology (CITEC), Bielefeld University, Bielefeld, 33619 Germany

**Keywords:** Clinical trial annotated corpus, Schematic annotation, Relational information extraction, Knowledge base population

## Abstract

**Background:**

The evidence-based medicine paradigm requires the ability to aggregate and compare outcomes of interventions across different trials. This can be facilitated and partially automatized by information extraction systems. In order to support the development of systems that can extract information from published clinical trials at a fine-grained and comprehensive level to populate a knowledge base, we present a richly annotated corpus at two levels. At the first level, entities that describe components of the PICO elements (e.g., population’s age and pre-conditions, dosage of a treatment, etc.) are annotated. The second level comprises schema-level (i.e., slot-filling templates) annotations corresponding to complex PICO elements and other concepts related to a clinical trial (e.g. the relation between an intervention and an arm, the relation between an outcome and an intervention, etc.).

**Results:**

The final corpus includes 211 annotated clinical trial abstracts with substantial agreement between annotators at the entity and scheme level. The mean Kappa value for the glaucoma and T2DM corpora was 0.74 and 0.68, respectively, for single entities. The micro-averaged *F*_1_ score to measure inter-annotator agreement for complex entities (i.e. slot-filling templates) was 0.81.The BERT-base baseline method for entity recognition achieved average micro- *F*_1_ scores of 0.76 for glaucoma and 0.77 for diabetes with exact matching.

**Conclusions:**

In this work, we have created a corpus that goes beyond the existing clinical trial corpora, since it is annotated in a schematic way that represents the classes and properties defined in an ontology. Although the corpus is small, it has fine-grained annotations and could be used to fine-tune pre-trained machine learning models and transformers to the specific task of extracting information about clinical trial abstracts.For future work, we will use the corpus for training information extraction systems that extract single entities, and predict template slot-fillers (i.e., class data/object properties) to populate a knowledge base that relies on the C-TrO ontology for the description of clinical trials. The resulting corpus and the code to measure inter-annotation agreement and the baseline method are publicly available at https://zenodo.org/record/6365890.

## Background

So far, there are few corpora in which clinical studies are manually annotated for the purpose of training information extraction models. Most of the available datasets provide coarse-grained annotations of the PICO elements^1^ only. The annotations are coarse-grained in that they typically provide text elements (at the phrase or sentence-level) to describe each PICO element. Therefore, these annotation schemes do not provide sufficient detail about the study design or how different arms, treatments (e.g. doses, duration, time of application, etc.), or other elements (e.g. quantitative results, size of effect, etc.) are interconnected. Thus, these annotation schemes are not rich enough to support the development of systems able to extract information from published clinical trials at a level of detail that is sufficient to support the comparison and aggregation of outcomes across multiple clinical trial publications.

To address this gap, in this work we describe the development of a corpus of clinical trials whose annotation scheme follows a structure derived from the C-TrO ontology [1], which has been designed to support the aggregation of clinical trials. In annotating the corpus, we distinguish between annotations at the level of entities and annotations at the level of complex classes or schemas. Annotations at the entity level comprise the markup of one **single non-decomposable** entity mentioned in the text. Examples of classes of such entities include publication year, clinical design (e.g., double-blind, multicenter), drug names, p-values, and more. Annotations at the level of complex classes comprise of a **slot-filler scheme** that represents more complex clinical concepts that can be decomposed into various typed slots (fields) that can be filled with the appropriate information. Examples of such complex clinical concepts are: interventional arms and the associated populations and interventions, medication protocols, defined endpoints and the related outcomes.

Besides annotating the slots of the complex clinical concepts that appear in a given publication, our corpus includes relations between the instances of the different complex concepts, e.g., relations between a specific treatment, the corresponding arm where the treatment was applied and the outcome for this pair of treatment/arm.

Aiming to provide a corpus that is annotated at the schema level as well as featuring relations between instances of the complex concepts, we implemented our annotation schema in an annotation tool designed for such schema-level annotation tasks on the basis of a given ontology. The entity annotations are exported and made available in a standard one-token-per-line format following the CoNLL format [2, 3], and the schema-level relational annotations are exported as RDF [4] triples (subject, predicate, object) following the C-TrO ontology.

The corpus is intended to support the development of information extraction (IE) systems that extract information from clinical trial publications at a level of comprehensiveness and detail that is needed to aggregate information across trials and that is not possible given the current state of the art in biomedical IE. Information extraction systems developed on the basis of our corpus can be evaluated at two levels: First, at the level of recognition of relevant entities and, second at the level of extracting a set of instantiated schemas that have relations to each other.

Existing corpora of clinical trials annotated to support clinical research typically include only PICO elements annotated in a coarse-grained fashion either at a sentence or phrase-level with the view that finer annotated entities or more detailed relevant information (e.g., age, sex, ethnicity) can be subsequently identified within these entities by automatic methods such as machine learning or rule-based models [5–7]. The identified data can then be used in specific tasks, ranging from answering clinical questions to the retrieval of relevant clinical documents.

One of the main uses of the sentence-level annotations of PICO elements has been to support the search for precise answers in an information retrieval setting from large medical citation databases such as PubMed [6]. Therefore, a search engine able to detect and index PICO elements in the text collection can help to retrieve relevant documents [8, 9]. While the coarse-grained sentence-level annotations are sufficient to support search for documents, they certainly do not suffice to support the aggregation of evidence, which requires annotations at a fine-grained level going beyond the current practices in annotation of PICO elements including their relationships to other PICO elements. In this respect, there is a small number of corpora that explicitly annotate relations between PICO elements, see [10–13]. The corpus created by Summerscales et al. [10] contains 263 RCT abstracts from the British Medical Journal (BMJ). The PICO elements are annotated in a coarse-grained manner by way of XML tags in which the intervention groups and their outcomes are related through IDs included as attributes in the related XML elements. The type of outcome, i.e. good or bad (adverse effects), is captured via XML attributes. Finer-grained elements (e.g. the descriptions of the intervention groups and the quantities associated with the outcomes) were automatically extracted from the coarse-grained annotated elements and then used to calculate summary statistics, such as the absolute risk reduction. Trenta et al. [11] describe a corpus of 99 hand-annotated clinical trial abstracts on glaucoma in which patient groups, arms, primary outcome descriptions, and results are tagged. In contrast to our ontology-based slot-annotations that consider complex relationships among atomic and composed entities and all types of endpoints and adverse effects, this corpus contains only simple relationships between the arms and their interventions through their tag names, which are formed by the abbreviation of the entity name and a number (e.g. *<a1 >:arm1 is related to <r1 >:result1*). The main use of this corpus is the training of information extraction systems that can extract evidence tables from text. Zlabinger et al. [12] created a corpus in which the PICO elements are annotated at both the phrase- and sentence-level by expert and non-expert annotators. Besides, a sub-set of the corpus includes the annotations of the sentiments of the results of the compared interventions. If an intervention is better than its comparator (e.g., more effective, less adverse effects), the sentiment is positive, otherwise negative. The corpus consists of 1,750 annotated RCT abstracts, of which 1,400 abstracts include sentiment labels. However, this corpus does not contain annotations of the relationships between PICO entities. Nye et al. [13] present the EBM-NLP corpus that was annotated through crowd-sourcing. The corpus is composed of around 5,000 RCT abstracts with the aim to facilitate the development of an information extraction model that supports the extraction of key evidence from RCT abstracts. The annotations include text spans that describe the PICO elements (e.g., age, type of intervention, sample size) at finer detail. The annotated entities are also mapped onto the medical vocabulary MeSH. However, the relationships between PICO elements are not annotated.

There are useful annotations provided by the described corpora for the aggregation of clinical trial evidence. For example, the sentiment annotations can be used when comparing treatment superiority in terms of efficacy and safety when aggregating several clinical trials results, while the mapping of entities onto a medical vocabulary is also useful for the normalization of terms. However, most corpora have been annotated at a coarse-grained level and to not contain crucial information about the clinical trials such as the risk of bias, the overall design of the clinical trial, or meta-data such as the country in which the study was conducted. Only the EBM-NLP corpus contains some of these finer-grained PICO elements. In contrast, the corpora of Summerscales et al. [10] and Trenta et al. [11] provide limited relationships between the PICO elements.

The population of knowledge bases with information extracted from scientific publications has received substantial interest. Different information extraction systems have been applied for this purpose, among them, named entity recognition, disambiguation or normalization (i.e. mapping of relevant entities to specialized vocabularies), and relation extraction to identify semantic relations between entities.

The main use of our corpus is to support the training of information extraction systems that can extract relevant information from published clinical trials to populate a knowledge base following the C-TrO ontology for the description and aggregation of clinical trial results. Current corpora do not consider the complex relationships necessary to populate a knowledge base and do not provide the basis for automatic extraction and comparison/aggregation of results across clinical trials. The extraction of information from clinical trials at a sufficient level of detail is however key to progress on the automatic generation of systematic reviews that is a central concern for the medical community [14].

## Methods

### Selection of texts for the corpus

In order to form the corpus, we searched for abstracts on glaucoma and type 2 diabetes mellitus (T2DM). We selected only two diseases to facilitate the task of annotators, who were not medical experts, to become familiar with the clinical concepts (e.g., endpoints and outcomes) used particularly for these two diseases.

The selected abstracts were required to follow the Consolidated Standards of Reporting Trials (CONSORT) [15], which recommend that authors include in the abstracts the compressed structure of the corresponding full article, including background, objective, methodology, results, and conclusions. These sections should comprise of the corresponding relevant elements (e.g. population and preconditions in methodology).

Then, we searched for relevant publications written in English using PubMed’s search tool PICO linguistics [16] and other search engines like Kopernio and Google search. The search filters used in PICO linguistics were the diseases and the drugs commonly used to treat the diseases in question, and publication types, such as clinical trials, systematic reviews, and meta-analysis articles. We took the abstracts from the retrieved clinical trials and the abstracts of the studies cited in the systematic reviews and meta-analysis to form our corpus. We excluded those abstracts with insufficient or unclear information on the study methodology and results, and those that did not follow the CONSORT recommendations.

Thus, the final corpus comprises of 211 abstracts, of which 107 are on glaucoma and 104 on type 2 diabetes mellitus (T2DM). All abstracts come from PubMed clinical trials that are randomized, are in phase 3 or 4, and compare at least two drug intervention arms. The abstracts were automatically downloaded from PubMed in text format and then tokenized and uploaded to the annotation tool.

### Annotation schema

**Fig. 1:**
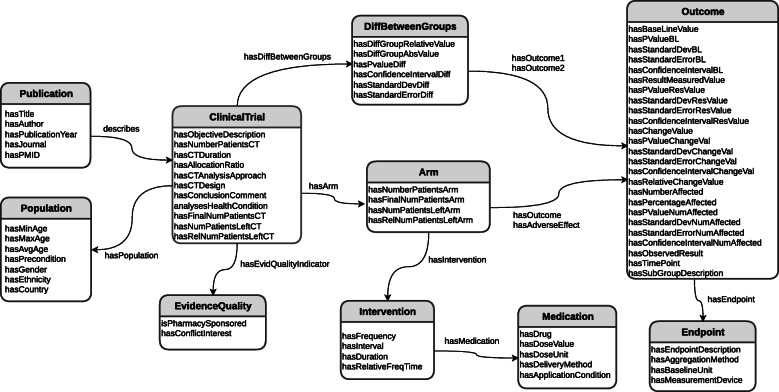
Annotation scheme based on C-TrO. The annotation schema follows the C-TrO structure with some variants to facilitate the annotation process

The annotation schema was derived from our clinical trial ontology C-TrO [1] that was designed to model fine-grained information of clinical trials. C-TrO covers the PICO elements which are the main components of a clinical trial. Furthermore, it contains other important elements for the aggregation and meta-analysis of clinical studies such as the numeric value of the change from baseline caused by the interventions, the statistical information about such changes, the baseline values, etc. In addition, this schema facilitates the required complex annotation task and considers the different ways in which the authors of clinical trial articles may report their methods and results. For example, authors can report their results either in terms of the amount of change from baseline, the measurement obtained at the last time point, or as a textual description. The schema based on C-TrO used for the annotation process is depicted in Fig. 1.

Then, the annotation task consists of annotating the classes and data/object properties contained in the C-TrO schema. In ontological terms, data properties relate individuals (i.e., instances of a given class) to literal data (e.g., strings, numbers, etc.), while object properties relate individuals to other individuals.

**Fig. 2:**
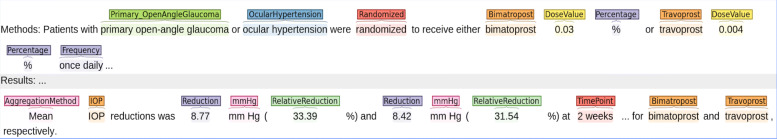
Example of the annotation of single entities according to the C-TrO schema. (Excerpt taken from abstract PMID 21034177 and annotated with INCEpTION [24] for illustrative purposes)

**Single entities** are **atomic entities** that can be individuals (i.e., instances of a class) or literal values, as defined by the underlying ontology. Figure 2 shows individual entities annotated according to our schema, in the comparison between the *bimatoprost* and *travosprost* interventions. For example, *Mean* is an individual of the AggregationMethod class^2^. In this example, *Mean* refers to the average value of the resulting IOP measurements in each arm^3^. The *Reduction* label indicates that the annotated number is the amount of IOP reduced from the baseline (i.e., the direction of the IOP change). The *Reduction* label allows to consider different ways in which the authors of the clinical trials may report such a reduction, as for example, a description (e.g. “the IOP reduction was 8.77 units”) or only the sign “-”. DoseValue is the annotation of the literal value *0.03* that denotes the dose of the administrated drug.

**Fig. 3:**
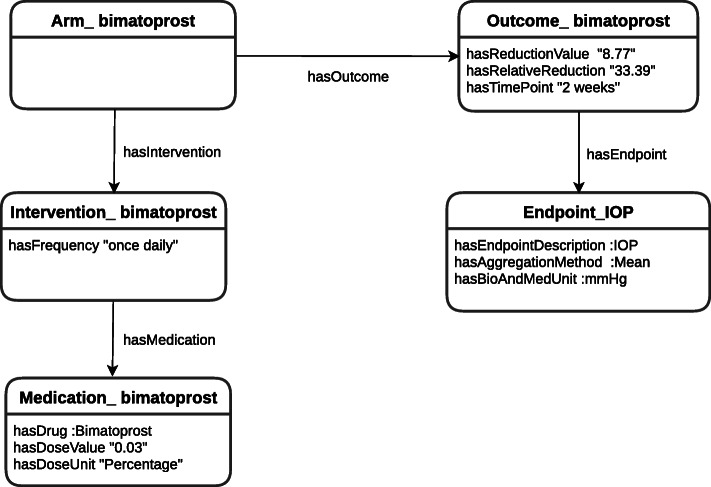
Schematic representation of the annotations of an interventional arm. The bimatoprost interventional-arm and its outcome, as reported in the excerpt of abstract PMID 21034177

**Complex entities** are composite entities consisting of several information items or slots. Following the example in Figs. 2, 3 depicts the schematic annotations referring to the *bimatoprost* intervention. The complex entities are the instances of the classes *Arm, Intervention, Medication, Endpoint and Outcome*. Intervention-bimatoprost is an instance of the *Intervention* class, which is composed of the single entity Frequency that is a literal value (“once daily”) and the relationship to the complex entity Medication-bimatoprost through the respective data property *hasFrequency* and object property *hasMedication*.

In RDF, the basic data modelling unit is a triple of the form 〈*s*,*p*,*o*〉 where *s* stands for subject *p* for predicate and *o* for object. In case of complex entities, the subject would represent an ID for the complex entity and the slots would be modelled as objects for different predicates. Multiple values for a subject/predicate are allowed in principle, unless the property is *functional*.

The following is an example of some of the triples that describe the entities and their relationships depicted in Fig. 3.

- _1_ 〈*data:Arm_bimatoprost rdf:type, ctro:Arm* 〉
- _2_ 〈*data:Arm_bimatoprost, ctro:hasIntervention, data:Intervention_bimatoprost* 〉
- _3_ 〈*data:Arm_bimatoprost, ctro:hasOutcome, data:Outcome_bimatoprost* 〉
- _4_ 〈*data:Intervention_bimatoprost, ctro:hasFrequency, “once daily”* 〉
- _5_ 〈*data:Intervention_bimatoprost, ctro:hasMedication, data:Medication_bimatoprost* 〉
- _6_ 〈*data:Medication_bimatoprost, ctro:hasDrug, data:Bimatoprost* 〉
- _7_ 〈*data:Medication_bimatoprost, ctro:hasDoseValue, “0.03”* 〉
- _8_ 〈*data:Medication_bimatoprost, ctro:hasDoseUnit, data:Percentage* 〉

Line 1 defines *Arm*_*bimatoprost* as an instance of class *Arm*. Lines 2 and 3 state that *Arm*_*bimatoprost* is related to instances of the type *Intervention* and *Outcome*, respectively. Line 4 and 5 describe that *Intervention*_*bimatoprost* is composed of frequency “once daily” which is a literal value, and the relation to an instance of type *Medication*. Lines 6 to 8 declare that *Medication*_*bimatoprost* is composed of the drug Bimatoprost which is an individual of type *Drug*, the dose unit Percentage which is an individual of type *ConcentrationUnit*, and the dose value that corresponds to the literal “0.03”.

Thus, the corpus is annotated both at the single-entity level as week as at the schematic level that includes complex entities and their relationships. The resulting annotations are provided in CoNLL format (atomic entities) as well as in RDF format (atomic and complex entities).

#### From classes and properties to template and slot-fillers

Text annotation according to the schema of a given ontology is challenging, as this type of annotation can be complex for annotators and curators since it involves the abstract conceptualization of text fragments that can correspond to class instances or data/objects properties of the ontology. To make the annotation process easier for annotators, we approached it as a template slot-filling task, where the templates represent composite classes and the slots represent their properties. The slots can only be filled with the appropriate type of entities according to the underlying ontology. Thus, the slot-fillers can be seen as the object (or range) of the properties (or relations) and the template as the subject (or domain).

**Fig. 4:**
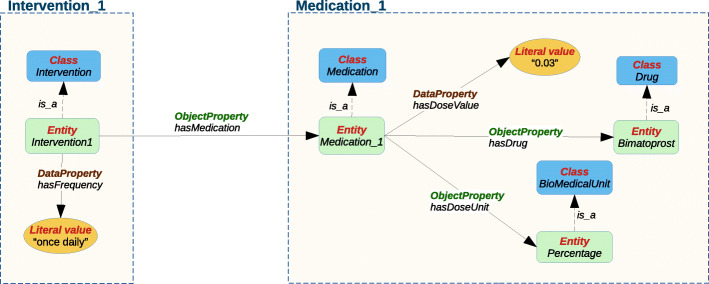
Classes and properties of the instances of the classes Intervention and Medication. (Intervention_1 and Medication_1, respectively)

Figure 4 shows a more detailed schema of some of the properties of Intervention_1 and Medication_1, which are instances of the complex classes Intervention and Medication, respectively. We can see that Intervention_1 has the data property *hasFrequency* and the object property *hasMedication*. The range of *hasFrequency* is “once daily”, which is a piece of text annotated as “Frequency” and is of literal type. The range of *hasMedication* is Medication_1 which is itself an instance of a composed class. Medication_1 has a data property that indicates a dose value, and two object properties *hasDrug* and *hasDoseUnit*. The entity Bimatoprost, which is an instance of the Drug class, is the range of *hasDrug*.

**Fig. 5:**
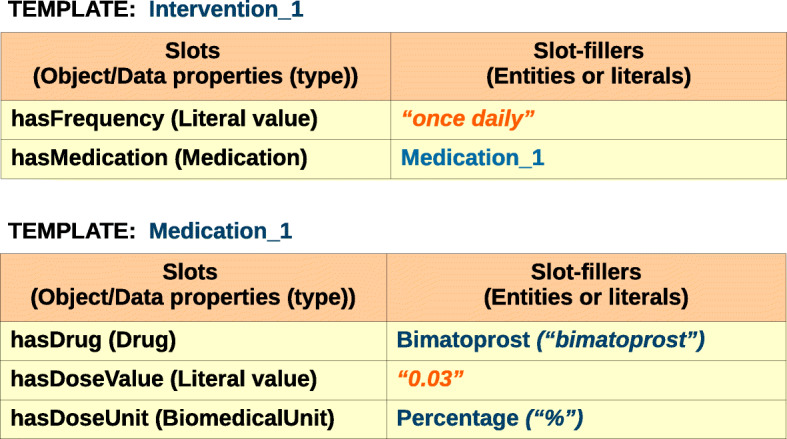
Templates and slot-fillers corresponding to the instances Intervention_1 and Medication_1. The texts in quotes are annotated text spans

Figure 5 shows the corresponding templates for the Intervention_1 and Medication_1 instances depicted in Fig. 4. For example, in the Intervention_1 template, the slot-filler for the *hasFrequency* slot is a literal value (e.g. “once daily”); the filler for the *hasMedication* slot is an instance of Medication type (e.g. Medication_1). In the Medication_1 template, the filler of the *hasDrug* slot is “Bimatoprost”, which is of Drug type.

The usage of templates facilitates the annotation task as the annotators only have to select a template type and fill in the slots by dragging and dropping the entities in question from the text without having to worry about the properties, classes, and subclasses. This is guided by the annotation tool on the basis of the underlying ontology schema.

Table 1 describes the annotation of single and complex entities in the context of text annotation, the ontology schema, and the template-filling approach.

**Table 1:** Description and examples of entities in text, ontology and template slot-filling

Context	Description	Examples
*Single (or atomic) entities*		
Annotation in text	Spans of text that refer to atomic entities	The text span “bimatropost” is annotated as Bimatropost. The text span “0.03” is annotated as DoseValue.
Ontology schema	Range of a dataProperty that is a *literal value* or - Range of an objectProperty that is an *individual* (atomic instance) of a given class	“0.03” is a literal representing a dose value and the range in the triple <Medication, *hasDoseValue*, DoseValue >. Bimatropost is an individual of class Drug and the range in the triple <Medication, *hasDrug*, Drug >
Template slot-filling	Slot-filler of the type specified by the corresponding ontology properties	Bimatropost (“bimatropost”) is the *hasDrug* slot-filler of a Medication template.
*Complex (or composed) entities*		
Annotation in text	*NA* (A complex entity is a composed entity and therefore it does not exist in the text. In the text there may be the entities that are elements of a complex entity.)	*NA*
Ontology schema	A complex class in the C-TrO ontology, and which is the domain of the corresponding dataProperties and objectProperties.	The Medication class, which is the domain in the triple <Medication, *hasDrug*, Drug >
Template slot-filling	Template of the type of the corresponding ontology class. The template’s slots correspond to the dataProperties and objectProperties of the class.	The Medication template, which has the *hasDrug* slot whose slot-filler must be an entity of Drug type.

#### The annotation tool SANTO

We used the ontology-driven slot-filling annotation tool SANTO [17], which is an intuitive tool that allows complex slot-filling and includes the relationships between different annotations following the conceptualization of a given ontology. SANTO is suitable for our annotation task since the slot-filling templates correspond to complex (or composed) entities, and a template has a set of property-slots (i.e., relationships) that can be filled with *a)* single entities which are annotated in the text or *b)* references to other complex entities (templates).

SANTO was configured according to the annotation schema based on C-TrO that was previously described. To annotate a single entity, the annotators first mark a span of text and then select the suitable label from a catalogue that is populated by the classes and individuals defined in the underlying schema. In order to annotate a complex entity, the annotators create a template and fill its slots with the appropriate annotated single entities or the references to other complex entities of the allowed types and according to the underlying schema. In SANTO, gold-standard annotations can be created through a curator account by merging the annotations of different annotators into a single file. The annotations can be exported into formats that enable interoperability with other data sources and tools, such as text files in which the span of text of the single entities and their annotation categories are indicated in CoNLL format, and as RDF triple files that contain the relationships.

### The annotation process

We first developed the annotation guidelines^4^ that include the definition of the general clinical trial terms and the specific terms for glaucoma and T2DM, and describe the annotation schema, the use of the annotation tool, and the way to annotate single and complex entities according to the schema. The guidelines were written by two authors and, in case of conflicts, these were resolved by consulting an expert in the medical field.

Our annotation team consisted of four annotators who were not experts in medical research, and an expert annotator. The expert annotator was one of the authors, who was familiar with medical terms and schematic annotation. The four annotators read the guidelines and participated in a pre-annotation training. We fine-tuned the guidelines according to the feedback received from the team and some of the authors. Although the four annotators were not medical experts, they achieved a good level of knowledge to perform the annotation work at the end of the training. In performing the actual annotations, half of the team annotated the glaucoma abstracts, and the other half the T2DM abstracts. The annotation was done in two phases.

**Phase 1.** Each abstract was first annotated with individual entities by two annotators, resulting in two annotated files. Afterward, each pair of annotated files was merged into a new file within SANTO. An expert annotator, the curator, accepted and merged both sets of annotations directly in those cases where the two annotators agreed. In case of disagreement between annotators, the curator resolved the disagreement. The curator could also remove incorrect annotations and add missing ones where necessary. The curation (or annotation adjudication) has proved to improve the annotation quality [18].

**Phase 2.** Subsequently, the curated texts were distributed among four annotators to annotate complex entities, that is, to create templates corresponding to the complex entities and fill their slots with the appropriate entities or references to other templates, as appropriate. The annotation of single and complex entities is described in the following^5^:

#### Annotation of single entities

Single entities are spans of text that are annotated as individuals of a given type or as literal values. The same entity can be expressed in text in different ways (e.g., uppercase or lowercase, or as an acronym). For example, “timolol”, “Timolol” or “Timolol Maleate” are different forms of the same entity (i.e., the drug timolol), and therefore are all annotated as *Timolol*.

**Fig. 6:**
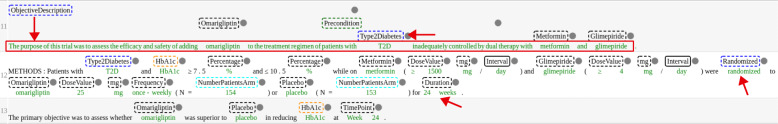
Example of single entities annotated with SANTO (abstract PMID 29110647). The red arrows indicate the annotated entities that are included in the complex entity in Fig. 8

A span of text can be annotated with the closest ancestor class if the corresponding individual (i.e., instance of a class) is not in the ontology and thus not in the annotation catalog. For example, if *Timolol* was not in the catalog, the annotator could choose the Drug label to annotate the text span “Timolol Maleate” because *Timolol* is an individual of the Drug class. Figure 6 shows single entities annotated with SANTO in a clinical trial on T2DM.

Notice that overlapping and embedded annotations are possible. Examples of these annotation cases are shown in Fig. 9, where sentence (a) has three overlapping *Timepoint* annotations, sentence (b) has two spans of text annotated as individuals of *Drug* that are embedded in an annotation of the same type (i.e., *Drug*). In sentence (c) there are three annotations embedded in an annotation of a different type (*ConclusionComment*).

#### Annotation of complex entities

The slot-filling templates that describe complex entities are pre-defined in SANTO according to the annotation schema. On this basis, the annotators instantiate templates whose slots are filled with single entities or references to other complex-entity templates. When a new template is created, the system gives it a default name. However, the annotator can change this name to one that gives a better indication of the content of the template. The template name becomes the identifier of, or reference to, the corresponding instance in the knowledge base.

Figure 8 depicts a template (complex entity) of ClinicalTrial type named “Clinical Trial 76755”, which is the default name given by the system. The ClinicalTrial template has single-entity and complex-entity slots.

Some of the single-entity slots are filled with the individual entities shown in Fig. 6. If there were several occurrences of the same entity in the text that fit the type of a single-entity slot, only one of them would be included in the corresponding slot, regardless of its position in the text.

The complex-entity slots in the template are for example *hasArm*, *hasDiffBetweenGroups*^6^, and *hasPopulation*. We can see, for example, that the *hasPopulation* slot contains a reference to the Population template (instance) “Population 76771”. Notice that SANTO allows to fill in slots only with entities that have types that are valid for the given slot according to the ontology, and constraints the number of slots of each type in a template according to the cardinality restrictions of the corresponding properties defined in the ontology. For example, 8 shows an example where there is more than one instance of the *Arm* template.

## Results

Table 2 shows the number of annotations for individual entities and major complex entities in each disease corpus and the joint corpus Glaucoma-T2DM. It can be seen that the number of annotations is almost balanced in the two disease corpora, except for the number of annotations of Endpoint and Result instances, which is higher in the T2DM corpus than in the glaucoma corpus. This may be due to the fact that T2DM studies typically include more endpoints compared to glaucoma studies, yielding a higher number of outcomes reported. It should be noted that the endpoints and their outcomes can refer to both primary and secondary outcomes, as well as adverse events.

**Table 2:** Number of annotations of single entities and main complex entities

Corpus	Glaucoma	T2DM	Glaucoma-T2DM
Single entities	10,685	11,704	22,389
Complex entities			
Arm	215	208	423
Intervention	241	215	456
Publication	107	104	211
Medication	259	232	491
ClinicalTrial	107	104	211
Endpoint	310	604	914
Outcome	620	1,098	1,718
DiffBetweenGroups	173	303	476
Total	2,032	2,868	4,900

**Fig. 7:**
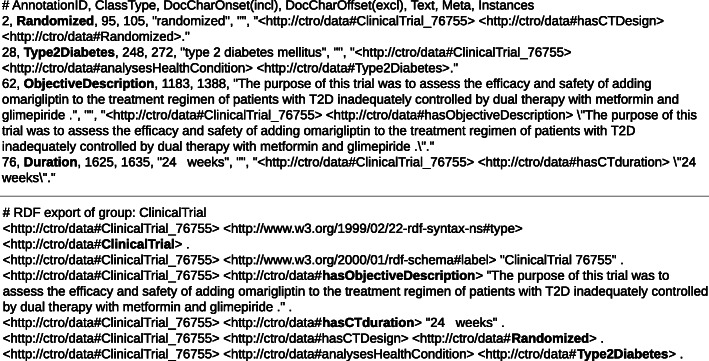
Example of annotations exported into CoNLL and RDF formats. The CoNLL-style file of entity annotations is at the top and the n-triple file of slot-template annotations is at the bottom

**Fig. 8:**
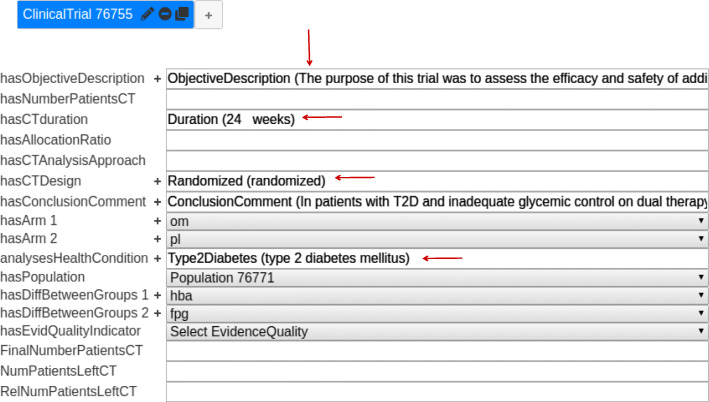
Example of the annotation of the complex entity ClinicalTrial with SANTO (abstract PMID 29110647). The red arrows indicate the single entities (see Fig. 6) that fill the corresponding slots. The slots *hasArm*, *hasPopulation* and *hasDiffBetweenGroups* contain references to other complex entities

To form the final corpus composed of both entity types, the complex entities (i.e., slot-fill templates) were exported to n-triple RDF format and the individual entities to CoNLL-style files. Figure 7 presents an example consisting of different files corresponding to the Annotations in Figs. 6 and 8, and which are part of the resulting corpus.

### Inter-annotation agreement

Inter-annotation agreement (IAA) helps to assess the reliability of the annotations of independent annotators over a corpus. A high IAA denotes that the annotation task has been well defined and that the annotations are consistent among annotators. Therefore, the annotations could be reproduced at other times and in similar contexts (e.g. other diseases). Thus, we calculated IAA for both single and complex entities as described in the remainder of this section.

#### Inter-annotator agreement on single entities

**Fig. 9:**
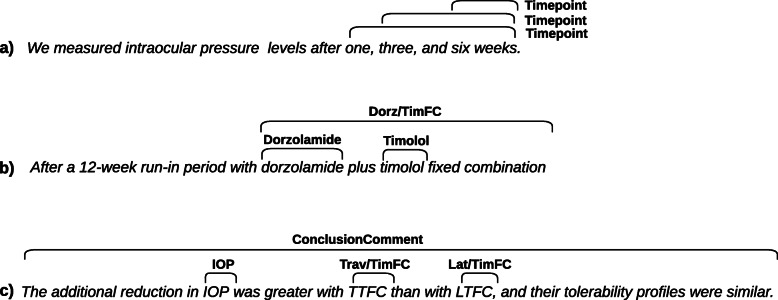
Example of annotations cases (excerpts from abstracts PubMed 8123096 and 23950156). **a** overlapping of annotations of the same type, **b** embedded annotation of the same type (i.e., *Drug*), and **c** embedded annotations of different types

As our corpus contains fine-grained annotated entities, the IAA considers cases such as partial and exact annotation matches, and overlapping and embedded annotations as the ones depicted in Fig. 9.

We rely on Cohen’s Kappa [19, 20] at the token level and for each annotation type that is accepted as slot-filler. Cohen’s Kappa is calculated as follows:

1 $$ Kappa=\frac{P(A) - P(E)}{1 - P(E)}  $$

Here, *P*(*A*) denotes the proportion of times that the two annotators agree, and *P*(*E*) is the proportion of times that it is expected they agree by chance. Kappa values lower than 0 indicate no agreement, 0-0.20 a slight, 0.21-0.40 a fair, 0.41-0.60 a moderate, 0.61-0.80 a substantial, and 0.81-1 an almost perfect agreement [21].

Since there are more than 300 categories of annotations, we grouped the annotations into the most general categories (i.e., ancestor classes), correspondingly. For example, *DisorderOrSyndrome* subsumes *Glaucoma* and *AngleClosureGlaucoma*. In addition to calculate Kappa for each annotation category, we also calculate the average Kappa for the whole corpus. As Table 3 shows, the average Kappa values for glaucoma and T2DM are *0.74* and *0.68*, respectively, denoting a substantial agreement. These results show that, although the clinical trials for glaucoma and T2DM differ in some of their characteristics, the IAA for both is substantial, suggesting that a good level of IAA could be reached in various disease contexts.

**Table 3:** Kappa values for the annotation of single entities. The hyphens indicate that no entities were annotated with the corresponding category

Annotation categories	Glaucoma	T2DM
AggregationMethod	0.89	0.41
AllocationRatio	0.91	0.00
Author	0.97	0.98
BaseLineValue	0.98	0.86
BioAndMedicalUnit	0.89	0.81
ChangeValue	0.96	0.77
ConclusionComment	0.96	0.92
ConflictInterest	0.00	0.00
Country	0.93	0.87
CTAnalysisApproach	0.26	0.19
CTDesign	0.85	0.86
DeliveryMethod	0.82	0.52
DiffGroupValues	0.71	0.78
DisorderOrSyndrome	0.93	0.98
DoseDescription	–	0.41
DoseValue	0.96	0.62
Drug	0.83	0.91
Duration	0.72	0.82
EndPointDescription	0.79	0.77
Ethnicity	0.83	0.86
Frequency	0.92	0.92
Gender	–	0.61
Journal	0.97	0.94
MeasurementDevice	0.19	0.00
ObjectiveDescription	0.83	0.88
ObservedResult	0.25	0.06
PMID	0.98	1.00
Precondition	0.47	0.66
PublicationYear	0.95	0.98
RelativeChangeValue	0.97	0.77
RelativeTime	0.32	0.71
ResultMeasuredValue	0.93	0.82
SubGroupDescription	0.23	0.46
TimePoint	0.46	0.71
Title	0.95	0.98
Avg. Kappa	0.74	0.68

Further, it can be seen in Table 3 that there is a high IAA on the annotation of entities that are frequently reported in clinical trials and that are related to the comparison of treatments, such as *EndPointDescription* (g=0.79, d=0.77), *Drug* (g=0.83, d=0.91), *DoseValue* (g=0.96, d=0.62), *ChangeValue* (g=0.96, d=0.77), *RelativeChangeValue* (g=0.97, d=0.77), and *ResultMeasuredValue* (g=0.93, d=0.82) among others. The kappa values for these annotations are mostly higher for glaucoma (g) than for T2DM (d).

**Causes of disagreement** To analyze the causes of disagreement on the annotations, we classify the annotated entities as numeric or textual. The numerical entities are, for example, the result values, p-values, etc., and the textual entities are the descriptions of preconditions, objective of the study, etc. The main source of disagreement in the annotation of textual entities was mainly due to the different limits of the length of the text assigned by each annotator. On the other hand, the disagreement on the annotations of numerical entities was lower than for textual entities. One of the most frequent causes of such disagreement was the exclusion/inclusion of a minus/plus sign in front of the annotated number. Another cause of disagreement was the annotation of homonymous entities. For example, when some annotators annotated the symbol “%” as a unit of concentration and other annotators as a rate value.

Templates with low agreement for both diseases are, for example, *ConflictInterest* (g=0, d=0), *MeasurementDevice* (g=0.19, d=0), and *ObservedResult* (g=0.25, d=0.06), which correspond to infrequent textual entities in abstracts. Future work will reveal if these slots are only frequent in our data sample or generally infrequent. The importance of annotating these entities will have to be determined depending on how crucial they are to support the use case of automatically aggregating evidence from multiple clinical trials.

#### Inter-annotator agreement on the annotation of complex entities

Measuring agreement on complex entities requires that the annotators agree on 1.) the number of instances of a given composed class and 2.) the semantic structure of these instances according to the underlying ontology. In terms of slot-filling templates, this means that the annotators should agree on the number of instances of complex templates, the relationships between them, and their slot-filler entities.

Since the calculation of the IAA on complex entities implies checking several elements, we selected a sample of 20 abstracts (i.e., around 10% of the total number of abstracts in the corpus) formed of 10 abstracts on glaucoma and 10 abstracts on T2DM that were slot-annotated by two different annotators. In this way, we could analyze the obtained IAA in more detail. The *F*_1_ score was used to evaluate the IAA on complex entities, considering that a *F*_1_ score of *1* represents a perfect agreement. The *F*_1_ score is the harmonic mean of precision and recall as defined in Eq. (2), where *tp* are true positives, *fp* false positives and *fn* false negative predictions.

2 $$  F_{1}= \frac{2tp}{fp + fn + 2tp}  $$

First, *F*_1_ is computed to compare the annotated entities assigned to single-entity slots of complex entities labeled by two different annotators.

In case there is more than one instance of the same complex-entity type in each annotation set, then *F*_1_ is calculated for the different combinations of instances to estimate the **best pairwise alignment**, i.e., the pair with the highest *F*_1_. Then, recall and precision are updated for the slots of the compared instances according to this alignment. Note that only single-entity slot-fillers are considered for computing the best alignment.

**Fig. 10:**
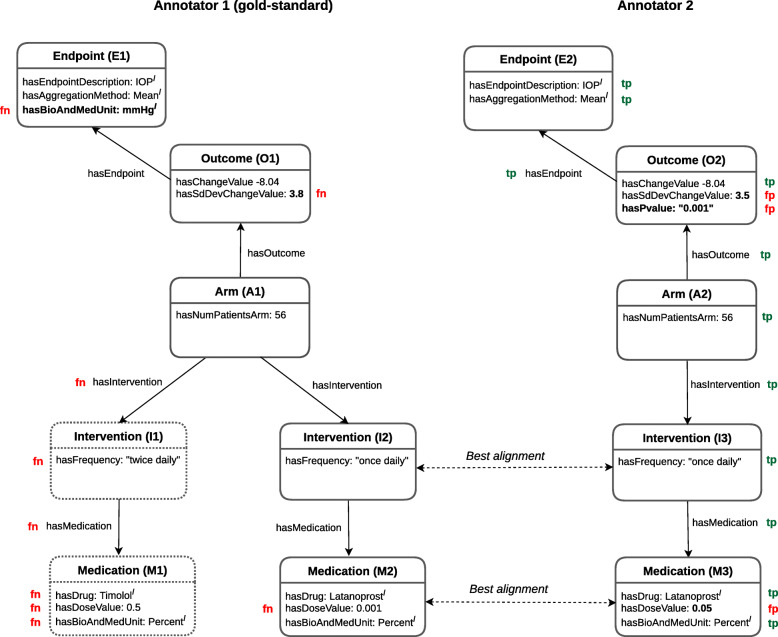
Example of the statistics for *F*_1_ to determine the IAA on complex entities between two annotators. *I*: the entity is an Individual. Otherwise, the entity is a literal value

Figure 10 depicts an example of the statistics (i.e., *tp*/*fp*/*fn*) for computing *F*_1_ to compare the complex-entity annotations of two annotators, where one of them is the gold-standard. If the single entities assigned to the slots of the pair of complex entities being compared match in type and value, then this counts as a true positive (*tp*). Otherwise, as a false positive (*fp*). When an annotation that exists in the gold-standard is missing, this counts as a false negative (*fn*).

The *F*_1_ scores for slots which have complex entities as slot-fillers are calculated using the previously computed best alignments. A pair of complex-entity slot-fillers is considered to be a *tp* if these slot-fillers have been aligned. For example, in the case presented in Fig. 10, *Annotator1* identifies two instances of the complex entity Medication called M1 and M2, and *Annotator2* identifies only one MedicationM3. Thus, there are two pairs: (M1, M3) and (M2, M3). *F*_1_ is calculated for each pair and the one with the higher *F*_1_ is selected. Assuming that (M2, M3) is the best alignment, then the *F*_1_ score for (M2, M3) is used to update the number of *tp*, *fp* and *tn*. Because M1 does not have a corresponding peer, then all its slot-filler entities are counted as *fp*.

The position of the annotations in the text is not evaluated, since a slot can be filled with any annotation that fulfills the allowed type for this slot, regardless of its position in the text. For example, in a given abstract there are two entities annotated as Insuline, one at position 5 and one at position 25. One annotator chooses the entity at position 5 to fill the *hasDrug* slot in an instance of Medication and another annotator chooses the entity at position 25. Both entities are appropriate for this slot.

Since the distribution of the number of slot-fillers with respect to the slot types is unbalanced, to measure the **overall agreement**, we used the micro-averaged *F*_1_ score, which allows weighting each prediction equally. The micro-average score is calculated from the true positives (*tp*), false positives (*fp*), and false negatives (*fn*) of the individual slot types. That is, the *tp*, *fp* and *fn* over all the slot types are summed up and inserted into Eq. (2). The overall agreement reached is *0.81* as shown in Table 4.

**Table 4:** *F*_1_ scores for the IAA on complex entities in the glaucoma-T2DM corpus of 20 abstracts. Slot-fillers that contain reference to other complex entities are in italics and single entity slot-fillers in normal font

Publication	*F*_1_	Population	*F*_1_
hasAuthor	0.87	hasCountry	0.84
hasTitle	1.00	hasAvgAge	1.00
hasJournal	0.97	hasMinAge	1.00
hasPublicationYear	0.95	hasPrecondition	0.86
hasPMID	0.97	hasMaxAge	1.00
*describes*	1.00	hasGender	1.00
ClinicalTrial	*F*_1_	DiffBetweenGroups	*F*_1_
hasNumberPatientsCT	1.00	hasDiffGroupAbsValue	1.00
analysesHealthCondition	0.78	hasDiffGroupRelValue	0.86
hasConclusionComment	0.96	hasConfIntervalDiff	1.00
hasFinalNumberPatientsCT	1.00	hasPvalueDiff	0.87
hasObjectiveDescription	1.00	hasStandardDevDiff	1.00
hasAnalysisApproach	1.00	*hasOutcome1*	0.65
hasCTDesign	0.98	*hasOutcome2*	0.59
hasCTduration	0.94		
*hasArm*	1.00		
*hasDiffBetweenGroups*	0.89		
*hasPopulation*	0.97		
Arm	*F*_1_	Intervention	*F*_1_
hasNumberPatientsArm	1.00	hasFrequency	0.95
*hasAdverseEffect*	0.32	hasRelativeFreqTime	0.94
*hasIntervention*	0.60	hasInterval	0.67
*hasOutcome*	0.60	*hasMedication*	0.69
Medication	*F*_1_	Endpoint	*F*_1_
hasDrug	0.83	hasEndoPointDescription	0.82
hasDeliveryMethod	0.40	hasBaselineUnit	0.81
hasDoseUnit	0.92		
hasDoseValue	0.89		
hasDoseDescription	0.86		
Outcome	*F*_1_	Outcome (cont.)	*F*_1_
hasBaselineValue	0.84	hasChangeValue	1.00
hasConfIntervalChangeValue	1.00	hasNumberAffected	1.00
hasObservedResult	0.81	hasPercentageAffected	0.93
hasPValueChangeValue	1.00	hasPValueResValue	1.00
hasRelativeChangeValue	1.00	hasResultMeasuredValue	1.00
hasSdDevChangeValue	1.00	hasSdErrorChangeValue	1.00
hasSdDevBL	0.93	hasSdDevResValue	1.00
hasSubGroupDescription	1.00	hasTimePoint	0.69
*hasEndpoint*	0.66		

Overall agreement: Micro-averaged *F*_1_ = 0.81

In Table 4, we can also observe that the *F*_1_ scores obtained for most of the single-entity slot-fillers of the complex entities range between *0.78* and *1.00*, denoting a high agreement. On the other hand, the lower *F*_1_ scores range from *0.32* to *0.69* for mostly the complex-entity slot-fillers, like *hasOutcome, hasMedication, hasEndpoint*, etc. This shows that the annotators disagree more on cross-referencing complex entities, i.e. when the slot-fillers refer to other complex entities than when the slot-fillers are single entities.

One of the causes of high IAA may be the fact that the slot-filling annotation was done on a corpus that contains curated annotations of single entities. On the other hand, we observe the following causes of disagreement:

i) The annotators miss to fill some slots,

ii) The annotators conceptualize the complex entities differently from what is stated in the guidelines. For example, this is the case when the annotators consider the treatments applied before randomization as part of the compared interventions rather than as part of the preconditions. For instance, in the following excerpt (abstract PMID 24237386) the drug “metmorfin” is part of the pre-condition since a criterion of eligibility for the clinical trial is that the participants have previously received a metformin treatment. However, the annotator created an intervention whose drug is metformin.

“Aim: This randomized, double-blind, placebo-controlled parallel-group study assessed the effects of sodium glucose cotransporter 2 inhibition by dapagliflozin on insulin sensitivity and secretion in subjects with type 2 diabetes mellitus (T2DM), who **had** inadequate glycemic control with *metformin*…”

Another example is when two drugs that are part of a fixed drug combination are mistakenly considered separately in two interventions, instead of in a single intervention. For example, in:

“Fixed-combination *brimonidine-timolol* versus latanoprost in glaucoma and ocular hypertension: a 12-week, randomized, comparison study.”

“brimonidine-timolol” should be annotated as the fixed drug combination *Brimo/TimFC* that belongs to a single intervention. Nevertheless, sometimes the annotators created two interventions for the same arm, one intervention for brimonidine and another for timolol.

### Baseline method for single entity recognition

We carried out the recognition of single entities both in abstracts and full-text articles in order to compare how a system trained on annotated abstracts performs on these two types of text.

We used a BERT-based approach. BERT (Bidirectional Encoder Representations from Transformers) [22] is a language representation model designed to pretrain deep bidirectional representations from unlabeled text. BERT has been pretrained on a vast amount of unannotated text that is, for example, available on the web. After pretraining, BERT can be fine-tuned on smaller datasets for specific NLP tasks or domains.

We used a pretrained BERT model on MEDLINE/PubMed abstracts^7^. We fine-tuned this model by adding two layers that predict the start and end positions of entities per entity type. If a token at position *p*_*s*_ in a given sentence is predicted to be the start token for an entity of type *t*, then the corresponding end token is given by the nearest token at position *p*_*e*_≥*p*_*s*_, which is predicted as the end position for the entity of type *t*. If there is no corresponding end token for a predicted start token, then the predicted start token is ignored. We trained the model with the Adam optimizer [23] for 30 epochs. We only consider entity types that occur at least 20 times in the respective training set. We report precision, recall, and *F*_1_ on the test sets with exact matching of entities. We consider a predicted entity in a given sentence to be correctly classified if there is an entity in the test set of the same type and the same start and end positions.

#### Entity recognition on abstracts

Table 5 shows the results of the recognition of single entities. We can observe that the micro average *F*_1_ scores are similar both for glaucoma and T2DM. The entities Drug and EndPointDescription obtained very low scores for glaucoma, while for T2DM these entities reached high scores. In the case of the Drug entity in glaucoma, the low scores may be due to the common presence of fixed combination drugs which are used as treatments for this disease. For example, in the fixed combination “brimozol/timolol”, brimozol and timolol are each annotated as single Drug entities, while brimozol/timolol is annotated as a single Drug entity that spans the two single Drug entities. It seems that the baseline method is not able to recognize this type of overlapping entity.

**Table 5:** Results of the single entity prediction with EXACT match on abstracts. The hyphens indicate that the entities do not appear in the respective datasets

	Glaucoma			T2DM		
Entity	Precision	Recall	*F*_1_	Precision	Recall	*F*_1_
Author	0.99	0.99	0.99	0.87	0.99	0.93
BaselineUnit	0.68	0.56	0.61	0.73	0.79	0.76
BaselineValue	0.91	0.67	0.77	0.88	0.75	0.81
CTDesign	0.79	0.89	0.84	0.87	0.91	0.89
CTduration	0.94	0.94	0.94	0.89	0.89	0.89
ChangeValue	0.97	0.85	0.90	0.73	0.90	0.80
ConclusionComment	0.79	0.79	0.79	0.00	0.00	0.00
ConfIntervalDiff	-	-	-	0.00	0.00	0.00
Country	0.82	0.95	0.88	0.91	0.56	0.69
DiffGroupAbsValue	0.73	0.89	0.80	0.86	0.60	0.71
DisorderOrSyndrome	0.97	0.92	0.94	0.64	0.47	0.55
DoseUnit	0.56	0.82	0.67	0.80	0.80	0.80
DoseValue	0.67	0.74	0.70	0.89	0.84	0.86
Drug	0.29	0.13	0.18	0.85	0.79	0.81
EndoPointDescription	0.17	0.11	0.13	0.68	0.77	0.72
Frequency	0.89	0.69	0.77	0.76	0.62	0.68
Journal	0.52	0.52	0.52	1.00	1.00	1.00
NumberAffected	0.71	1.00	0.83	1.00	0.87	0.93
NumberPatientsArm	0.81	0.81	0.81	1.00	0.87	0.93
NumberPatientsCT	0.93	0.93	0.93	0.93	1.00	0.97
ObjectiveDescription	0.58	0.48	0.52	0.38	0.28	0.32
ObservedResult	0.00	0.00	0.00	0.00	0.00	0.00
PMID	1.00	1.00	1.00	1.00	1.00	1.00
PValueChangeValue	0.55	0.75	0.63	0.64	0.64	0.64
PercentageAffected	0.86	1.00	0.93	0.98	0.94	0.96
Precondition	0.71	0.22	0.33	0.40	0.16	0.23
PublicationYear	1.00	1.00	1.00	1.00	1.00	1.00
PvalueDiff	0.46	0.61	0.52	0.85	0.92	0.88
RelativeChangeValue	1.00	0.67	0.80	-	-	-
RelativeFreqTime	0.44	0.67	0.53	-	-	-
ResultMeasuredValue	0.84	0.93	0.88	0.83	1.00	0.90
SdDevBL	1.00	0.80	0.89	0.71	0.45	0.56
SdDevChangeValue	1.00	0.67	0.80	0.55	0.86	0.67
SdDevResValue	0.83	1.00	0.91	0.37	0.86	0.52
SdErrorChangeValue	1.00	1.00	1.00	-	-	-
TimePoint	0.70	0.67	0.68	0.74	0.67	0.70
Title	0.93	0.82	0.87	0.91	0.77	0.83
Micro average:	0.80	0.73	0.76	0.81	0.73	0.77

We can also observe that, in general, entities that are long textual descriptions (e.g., ConclusionComment, ObservedResults and ObjectiveDescription) tend to get low scores with exact match. They may get higher scores with partial matching.

#### Entity recognition coverage on full-text articles

In order to see the performance of the baseline system fine-tuned with our abstract corpus on the task of recognizing entities with exact matches in full-text articles, we created a new test dataset. This dataset is composed of full articles that are freely available and correspond to some of the abstracts included in the test datasets for the previous experiment. The new test dataset is composed of 20 full-text articles, of which 13 articles are on T2DM and 7 on glaucoma. The abstracts, figures, tables, and references were removed from these files.

We used the fine-tuned BERT model on the full-text article dataset. We calculated the exact match by checking how many of the predicted entities were also tagged in the corresponding curated annotated abstracts (here called ground truth set). The results of this coverage are shown in Table 6.

**Table 6:** Results of the entity prediction with EXACT match on full text articles. The hyphens indicate that the entities do not appear in the respective datasets

	Glaucoma			T2DM		
Entity	Precision	Recall	*F*_1_	Precision	Recall	*F*_1_
Author	0.00	0.00	0.00	0.27	0.06	0.10
BaselineUnit	0.75	0.60	0.67	0.42	0.79	0.55
BaselineValue	0.11	0.25	0.15	0.24	0.45	0.32
CTDesign	0.59	0.73	0.65	0.50	0.87	0.63
CTduration	0.19	0.60	0.29	0.31	0.69	0.43
ChangeValue	0.02	0.20	0.04	0.16	0.36	0.22
ConclusionComment	0.05	0.08	0.06	0.00	0.00	0.00
ConfIntervalDiff	-	-	-	0.00	0.00	0.00
Country	0.14	0.33	0.20	0.00	0.00	0.00
DiffGroupAbsValue	0.00	0.00	0.00	0.03	0.06	0.04
DisorderOrSyndrome	0.53	0.83	0.65	0.75	0.75	0.75
DoseUnit	0.20	0.50	0.29	0.80	0.92	0.86
DoseValue	0.29	1.00	0.44	0.50	0.77	0.61
Drug	0.14	0.19	0.16	0.73	0.92	0.81
EndoPointDescription	0.14	0.50	0.22	0.20	0.54	0.29
Frequency	0.60	0.67	0.63	0.40	0.53	0.46
Journal	0.00	0.00	0.00	1.00	0.46	0.63
NumberAffected	0.00	0.00	0.00	0.02	0.50	0.04
NumberPatientsArm	0.00	0.00	0.00	0.34	0.65	0.45
NumberPatientsCT	0.25	0.75	0.37	0.21	0.50	0.30
ObjectiveDescription	0.00	0.00	0.00	0.00	0.00	0.00
ObservedResult	0.00	0.00	0.00	0.00	0.00	0.00
PMID	1.00	0.29	0.44	1.00	0.31	0.47
PValueChangeValue	0.00	0.00	0.00	0.00	0.00	0.00
PercentageAffected	0.00	0.00	0.00	0.14	0.77	0.24
Precondition	0.00	0.00	0.00	0.11	0.07	0.08
PublicationYear	0.60	0.43	0.50	0.90	0.69	0.78
PvalueDiff	0.21	0.33	0.26	0.15	0.37	0.21
RelativeChangeValue	0.04	1.00	0.08	-	-	-
RelativeFreqTime	0.20	0.50	0.29	-	-	-
ResultMeasuredValue	0.47	0.50	0.48	0.07	0.80	0.13
SdDevBL	0.11	0.25	0.15	0.44	0.36	0.40
SdDevChangeValue	0.00	0.00	0.00	0.00	0.00	0.00
SdDevResValue	0.46	0.75	0.57	0.05	0.71	0.09
SdErrorChangeValue	0.00	0.00	0.00	-	-	-
TimePoint	0.20	0.50	0.29	0.06	0.33	0.10
Title	0.00	0.00	0.00	0.00	0.00	0.00
Micro average:	0.18	0.31	0.23	0.20	0.44	0.28

The low scores obtained for the meta-information (i.e., Publication: Author, Title, Journal, PublicationYear, PMID, and Country) of the clinical trials for both diseases were mainly due to the different formats of the free full-text articles. For example, in the abstracts the format for author name is [surname(s) name initial(s)], while in the full-text is: [name(s) surname]. For instance, in the abstract PMID 27740719, the name of the first author is written as *“Shankar RR”*, while in the corresponding full text (PMCID: PMC5415484) is *“R Ravi Shankar”*. Because the system compares exact matches it considers these author names as mismatches.

Possible causes that the system did not find relevant information, such as baseline data, results values, and the difference between groups are: 1.) these data were included in figures or tables, which were eliminated; 2.) the baseline system could not adequately predict them as it was pretrained and fine-tuned with abstracts that have a different structure from that of full texts; 3.) our comparison was quite strict when comparing to exact matches. With partial matches, higher scores may be obtained.

We also tried a simple partial match, where a predicted entity was considered correct if there was an entity in the ground truth set with at least one overlapping token. Then, this entity in the ground truth set could not be used for any other subsequent alignment. The results in Table 7 with partial match show that the average precision scores for glaucoma and T2DM are similar to the ones reached with exact match in Table 6, while the average recall for both diseases increased.

**Table 7:** Results of the entity prediction on full text articles with PARTIAL match. The hyphens indicate that the entities do not appear in the respective datasets

	Glaucoma			T2DM		
Entity	Precision	Recall	*F*_1_	Precision	Recall	*F*_1_
Author	0.78	0.12	0.20	0.64	0.18	0.29
BaselineUnit	0.31	0.90	0.46	0.24	0.79	0.37
BaselineValue	0.10	0.25	0.14	0.31	0.60	0.41
CTDesign	0.42	0.77	0.55	0.40	0.97	0.56
CTduration	0.17	0.80	0.29	0.37	0.92	0.53
ChangeValue	0.08	0.80	0.15	0.31	0.75	0.44
ConclusionComment	0.52	0.92	0.67	0.00	0.00	0.00
ConfIntervalDiff	-	-	-	0.00	0.00	0.00
Country	0.10	0.33	0.15	0.00	0.00	0.00
DiffGroupAbsValue	0.27	0.80	0.40	0.37	0.82	0.51
DisorderOrSyndrome	0.34	0.92	0.50	0.67	0.83	0.74
DoseUnit	0.14	1.00	0.25	0.46	1.00	0.63
DoseValue	0.15	1.00	0.27	0.44	0.92	0.60
Drug	0.33	0.56	0.42	0.51	0.96	0.67
EndoPointDescription	0.10	0.50	0.16	0.24	0.93	0.38
Frequency	0.25	0.67	0.36	0.29	0.73	0.42
Journal	1.00	0.29	0.44	0.86	0.46	0.60
NumberAffected	0.00	0.00	0.00	0.02	0.50	0.04
NumberPatientsArm	0.00	0.00	0.00	0.30	0.65	0.41
NumberPatientsCT	0.23	0.75	0.35	0.20	0.50	0.29
ObjectiveDescription	1.00	0.20	0.33	1.00	0.08	0.15
ObservedResult	0.00	0.00	0.00	0.50	0.33	0.40
PMID	1.00	0.29	0.44	1.00	0.31	0.47
PValueChangeValue	0.22	1.00	0.36	0.00	0.00	0.00
PercentageAffected	0.00	0.00	0.00	0.14	0.90	0.25
Precondition	1.00	0.17	0.29	0.56	0.33	0.42
PublicationYear	0.60	0.43	0.50	0.75	0.69	0.72
PvalueDiff	0.37	0.67	0.48	0.23	0.97	0.38
RelativeChangeValue	0.04	1.00	0.07	-	-	-
RelativeFreqTime	0.13	1.00	0.24	-	-	-
ResultMeasuredValue	0.59	0.62	0.61	0.08	0.93	0.14
SdDevBL	0.11	0.25	0.15	0.56	0.45	0.50
SdDevChangeValue	0.00	0.00	0.00	0.00	0.00	0.00
SdDevResValue	0.46	0.75	0.57	0.04	0.71	0.08
SdErrorChangeValue	0.00	0.00	0.00	-	-	-
TimePoint	0.17	0.75	0.28	0.05	0.83	0.10
Title	0.00	0.00	0.00	0.00	0.00	0.00
Micro average:	0.22	0.50	0.30	0.22	0.63	0.33

Notice that a more complex partial matching method that considers overlapping of entities of the same type and embedded entities of the same and different type would give more precise results that the one used.

## Discussion

Our final corpus of 211 clinical trial abstracts of glaucoma and T2DM clinical trials obtained a substantial inter-annotator agreement at the entity and schema levels.

Due to the high level of detail of the annotations, the calculation of the inter-annotation agreement on single entities considers perfect and partial matches, as well as embedded and overlapping annotations. Furthermore, it considers as an agreement when the same entity is annotated either with a specific or a general category. The high agreement reached in most of the annotations of single and complex entities may also be due to the provision of clear annotation guidelines and effective training of the annotators. However, barriers for the annotation task are the inherent complexity of the medical domain and the different ways in which the authors of the studies describe their methodology and results in the limited space of the published abstracts. For example, sometimes the authors only report the difference between intervention groups, the last time point measurement, or the amount of change in the measurements from the baseline, and omit other relevant information. Compared to the clinical trial information extracted by Trenta et al. [11] which aims to complete evidence tables, our corpus would support the development of information extraction systems that can extract more detailed information akin to more complete trial result reports commonly included in clinical trial articles (such as baseline characteristics, results, and adverse effects). For example, in Trenta et al. [11] the information extracted does not include the dose of the drug administered, the baseline measurements, the average age of the patients, the number of participants in each arm, the adverse events, etc. that are part of these tables. Furthermore, with simple SPARQL queries on the RDF files generated from the curated (i.e. gold-standard) annotated corpus, it would be possible to generate such evidence tables on results and adverse effects.

To present the use of our corpus, we applied a BERT-based system to recognize single entities both in abstracts and in full-text articles. For abstracts, the method achieved micro- *F*_1_ scores of *0.76* for glaucoma and *0.77* for T2DM with exact matching, which is a good performance as a baseline.

However, for full texts the baseline method achieved very low scores with exact matching for the prediction of most entities. We have pointed out that the main reason for this is due to the different text format in which the baseline method was pretrained and fine-tuned (i.e., using PubMed abstracts).

Furthermore, we found that it is not always convenient to annotate complete texts, since they commonly have a more complex structure and make annotation a long and painful process. Also, it is possible that important information is still missing in the full texts, since this may be contained in tables/figures or in other documents, such as supplementary material, protocol documents or registration records. On the other hand, since the annotated abstracts follow the CONSORT structure, they summarize the corresponding articles quite well, contain the relevant information for the aggregation of clinical trials, and are easier to annotate in both entity and schematic levels.

On the other hand, the prediction of complex-entity slot-fillers is still a challenge for future work. Nevertheless, the current baseline methods results are encouraging and demonstrate that our fined-grained corpus may be useful for training systems that extract schematic information from clinical trial abstracts that are beyond coarse-grained single PICO entities.

## Conclusions

In this work, we presented a corpus composed of 104 T2DM abstracts and 107 glaucoma abstracts. The corpus contains annotations for both single and complex entities, as well as their relationships. The corpus is delivered in RDF format for the complex entities (i.e., schematic annotation) and in CoNLL-style for single entities. We have obtained significant inter-annotation agreement for both kinds of annotations. We carried out an analytical process and discussions with physicians and annotators in order to get a consensus about the most suitable way to annotate each abstract. In these discussions, we developed an annotation schema that attempts to keep the original C-TrO structure and at the same time captures the different ways in which authors of published clinical studies may express their methodology and results. To reach a clear consensus has not been easy, considering that the annotated data is meant to be used for training of an information extraction system that identifies simple and complex entities and their relationships in order to populate a knowledge base.

Despite the relatively small size of the corpus, it has been shown to be useful in fine-tuning a baseline NER system and, due to its detailed level of annotations, the system achieved encouraging accuracy in abstracts. Working with abstracts instead of full texts has several advantages. First, the task of annotating abstracts involves less effort compared to annotating full texts. Second, most existing deep learning methods including transformers are pre-trained on the basis of abstracts and have a better performance on these.

In future work, the corpus will be used in information extraction systems that in addition to improving the performance of the presented baseline method, recognize the slot-fillers of complex entities, and the cross-references to other complex entities.

## Data Availability

The corpus of clinical abstracts, annotation guidelines, and annotated abstracts, as well as the source code of the implementation of the baseline method, inter-annotation agreement and tokenization are publicly available at https://zenodo.org/record/6365890.

## Abbreviations

EBM: evidence-based medicine
IAA: inter-annotator agreement
IE: information extraction
KB: knowledge base
NER: named entity recognition
NLP: natural language processing
RCT: randomized clinical trial
RE: relation extraction
RDF: resource description framework
T2DM: Type two diabetes mellitus

## Footnotes

1 PICO stands for Patients/Population, Intervention, Control/Comparison and Outcome

2 AggregationMethod refers to the method by which the results of the intervention are aggregated.

3 Arm refers to each group of participants in a clinical trial that receives an interventions (or a placebo).

4 The annotation guidelines can be accessed in the repository mentioned in the “Availability of data and materials” section.

5 For more details about the annotation process, the reader can consult the annotation guidelines in the repository mentioned in the “Availability of data and materials” section.

6 hasDiffBetweenGroups indicates the difference between the outcomes of the two interventions being compared.

7 https://tfhub.dev/google/experts/bert/pubmed/2
